# Long-Term Mortality in Patients Diagnosed with Meningococcal Disease: A Danish Nationwide Cohort Study

**DOI:** 10.1371/journal.pone.0009662

**Published:** 2010-03-12

**Authors:** Casper Roed, Lars Haukali Omland, Frederik Neess Engsig, Peter Skinhoj, Niels Obel

**Affiliations:** Department of Infectious Diseases, Rigshospitalet, University of Copenhagen, Copenhagen, Denmark; Singapore Immunology Network, Singapore

## Abstract

**Background:**

In contrast to the case fatality rate of patients diagnosed with meningococcal disease (MD) the long-term mortality in these patients is poorly documented.

**Methodology/Principal Findings:**

We performed a nationwide, population-based cohort study including all Danish patients diagnosed with MD from 1977 through 2006 and alive one year after diagnosis. Data was retrieved from the Danish National Hospital Register, the Danish Civil Registration System and the Danish Register of Causes of Death. For each patient four age- and gender-matched individuals were identified from the population cohort. The siblings of the MD patients and of the individuals from the population cohort were identified. We constructed Kaplan-Meier survival curves and used Cox regression analysis, cumulative incidence function and subdistribution hazard regression to estimate mortality rate ratios (MRR) and analyze causes of death. We identified 4,909 MD patients, 19,636 individuals from the population cohort, 8,126 siblings of MD patients and 31,140 siblings of the individuals from the population cohort. The overall MRR for MD patients was 1.27 (95% confidence interval (CI), 1.12–1.45), adjusted MRR, 1.21 (95% CI, 1.06–1.37). MD was associated with increased risk of death due to nervous system diseases (MRR 3.57 (95% CI, 1.82–7.00). No increased mortality due to infections, neoplasms or cardiovascular diseases was observed. The MRR for siblings of MD patients compared with siblings of the individuals from the population cohort was 1.17 (95% CI, 0.92–1.48).

**Conclusions:**

Patients surviving the acute phase of MD have increased long-term mortality, but the excess risk of death is small and stems mainly from nervous system diseases.

## Introduction

The case-fatality rate of meningococcal disease (MD) is well documented and reported to be approximately 10% in the developed world and substantially higher in non-industrialized countries [1]–[3]. In contrast, long-term mortality in this patient population is poorly documented [4].

A recent study from Bristol indicated that the incidence of MD was increased in areas of social deprivation [5]. Therefore studies are needed, not only to establish whether MD patients suffer from increased long-term mortality, but also to address to what extent this potentially increased mortality could be explained by family related risk factors.

We performed a nationwide, population-based cohort study to determine whether patients surviving the first year after a MD diagnosis have increased mortality compared with an age- and gender-matched population cohort. Further, we determined the specific causes of death. As a measure of unaccounted for confounders, we estimated the mortality in siblings of MD patients compared with siblings of individuals from the population cohort.

## Methods

### Ethics Statement

The study was approved by the Danish Data Protection Agency. The data was deidentified and anonymised at source.

### Setting

The population of Denmark on January 1, 2008, was 5.5 million inhabitants [6]. Over the last decades there has been a declining incidence rate of MD in Denmark (1987: 5.8/100,000, 1997: 4.5/100,000 and 2007: 1.4/100,000) [7], [8].

### Data Sources

We used the unique 10-digit Central Person Registration number (CPR number), assigned to all Danish citizens at birth or immigration to track individuals in the following registers and to avoid multiple registrations.

The Danish National Hospital Register was initiated in 1977 and contains information on all patients discharged from Danish hospitals. Records for each inpatient admission include CPR number, hospital department, dates of admission and discharge diagnosis coded according to the International Classification of Diseases 8^th^ (ICD-8) and 10^th^ (ICD-10) revision [9]. From this register, we extracted date of MD diagnosis, along with data on inpatient admissions prior to MD diagnosis.

The Danish Civil Registration System was established in 1963 and contains demographic data and vital status of all Danish citizens [10]. We extracted data on birth, gender, date of immigration and emigration, loss to follow-up, date of death and identity of siblings.

The Danish Register of Causes of Death contains information from all Danish death certificates since 1943 and is complete through 2006. Causes of death are coded according to ICD-8 and ICD-10, and registered as primary (immediate cause of death), secondary or tertiary cause of death [11]. From this register we extracted specific causes of death as recorded as the primary cause of death.

### Study Population

MD patients: From the Danish National Hospital Register, we identified all patients who were registered during the period January 1, 1977, to December 31, 2006, for the first time with a diagnosis of MD, ICD-8 codes: 036.09–036.99 and ICD-10 codes: A39.0–A39.9. Patients were excluded in case they died, emigrated or were lost to follow-up within the first year after the diagnosis of MD, and in case they were diagnosed with other neuroinfections (as specified in Appendixes S1) prior to MD, did not live in Denmark at the date of the MD diagnosis or were born outside Denmark. The index date of these individuals was defined as one year after the date of first MD diagnosis.

Population cohort: From the Danish Civil Registration System we identified four individuals for each MD patient matched on gender and date of birth, all of whom were born in Denmark, alive and living in Denmark at the index date of the corresponding MD patient.

Cohort of siblings: From the Danish Civil Registration System, we identified all registered full and half-siblings for both MD patients and individuals from the population cohort. Siblings born before January 1, 1952 were excluded in order to reduce selection bias, as only a small fraction of individuals born before 1952 had siblings registered in the Danish Civil Registration System, but thereafter the registration is more than 98% complete [12].

### Outcome

The primary study outcome was time from index date to death. The secondary outcome was time from index date to date of specific cause of death. The specific causes of death were categorized by ICD codes as listed in Appendixes S1.

### Statistical Analysis

Time was calculated from index date to date of death, emigration, loss to follow-up or January 1, 2008, which ever came first. In the analyses of cause specific mortality time was censored at January 1, 2007, as the Danish Register of Causes of Death was complete through 2006. Kaplan-Meier analyses were used to construct survival curves. Cox regression analyses were used to estimate mortality rate ratios (MRR) adjusted for calendar periods and for inpatient admission in the period of two years prior to the date of MD diagnosis. Calendar periods for the date of MD diagnosis were introduced as design variables grouped in the three time periods 1977–1986, 1987–1996, and 1997–2006. We adjusted for inpatient admissions during the two years prior to the date of MD diagnosis using (a) the total number of inpatient days as a continous variable and (b) a series of 18 indicator variables for the primary discharge diagnoses, as listed in Appendixes S1.

We assessed the proportional hazards assumption with plots and tests that were based on smoothed scaled Schoenfeld residuals and cumulative residuals. We stratified calculations of overall mortality by gender, age at diagnosis (0-<15, 15-<30, 30-<60 and 60 years or older) and type of MD (meningococcal meningitis or acute meningococcemia as primary diagnosis). We further calculated MRRs for the first 15 years after index date and for the period 16–30 years after the index date. A sub-analysis calculating the MRRs was performed, in which patients diagnosed with nervous system or genitourinary diseases in the first year after the MD diagnosis were excluded.

In a robustness analysis, using a Cox regression model, adjusted for age and gender we included only patients who had not been admitted to hospital in the period of two years prior to the date of MD diagnosis.

We computed the cumulative incidence of specific causes of death, taking into account that these were competing risks and used competing risks regression to estimate cumulative incidence of specific causes of death [13], [14].

We assessed the long-term mortality of the siblings in accordance with a previous study [12]. For siblings, time was calculated from one year of age of the sibling (to exclude peri- and postnatal mortality) or index date of the corresponding study participant, which ever came last, until date of death, emigration, loss to follow-up or January 1, 2008. MRR was calculated for siblings of MD patients compared with siblings of individuals from the population cohort and adjusted for calendar periods (design variable), age (design variable grouped in the age-intervals 1-<15, 15-<30, 30-<60 and 60 years or older at index date) and gender. As only siblings born after January 1, 1952 were included in these analyses, we calculated the MRR for MD patients compared with individuals from the population cohort born after January 1, 1952 for comparison. SPSS version 15.0 (SPSS Inc., Chicago, Il, USA) and R software, version 2.8.1 were used for data analysis.

## Results

### Baseline Characteristics

We identified 5,356 patients diagnosed with MD in the period 1977–2006. Within the first year of diagnosis of MD, 445 (8.3%) patients died, one emigrated and one was lost to follow-up leaving a total of 4,909 patients and 19,636 individuals from the population cohort in the study. 62% of the patients included in the study were diagnosed before they were 15 years of age. During the period of two years preceding the date of MD diagnosis the MD patients had a significantly higher admission rate for e.g. infectious diseases, neoplasms and nervous system diseases (Table 1).

**Table 1:** Characteristics of meningococcal disease (MD) patients and individuals from the population cohort.

	Patients with MD*	Individuals from the population cohort*	P-value
Number of study participants	4,909	19,636	
Males	2,661 (54.2)	10,644 (54.2)	
Age, median (years, IQR)	8.9 (2.3–17.8)	8.9 (2.3–17.8)	
Age at MD diagnosis			
0–14 years	3,070 (62.5)	12,280 (62.5)	
15–29 years	1,106 (22.5)	4,424 (22.5)	
30–59 years	473 (9.6)	1,892 (9.6)	
60+ years	260 (5.3)	1,040 (5.3)	
Primary diagnosis			
Meningococcal meningitis	3,297 (67.2)		
Acute meningococcaemia	1,211 (24.7)		
Chronic meningococcaemia	23 (0.5)		
Waterhouse Friderichsen syndrome	11 (0.2)		
Other specified conditions	367 (7.5)		
Calender period at MD diagnosis			
1977–1986	1,513 (30.8)	6,052 (30.8)	
1987–1996	2,089 (42.6)	8,356 (42.6)	
1997–2006	1,307 (26.6)	5,228 (26.6)	
Observation time (years)	73,058	293,585	
Emigration during study period	114 (2.3)	538 (2.7)	
Lost to follow-up during study period	2 (0.04)	5 (0.03)	
Number of study subjects with inpatient admission in the period of two years prior to the date of MD diagnosis	1,753 (35.7)	6,462 (32.9)	<0.001
Patients admitted with the following diagnosis-categories in the period of two years prior to the date of MD diagnosis			
Infectious diseases	109 (2.2)	242 (1.2)	<0.001
Neoplasms	34 (0.7)	90 (0.5)	0.04
Blood/immune diseases	11 (0.2)	22 (0.1)	0.06
Endocrine diseases	27 (0.6)	78 (0.4)	0.14
Mental disease/drug abuse	19 (0.4)	46 (0.2)	0.06
Nervous system diseases	36 (0.7)	74 (0.4)	0.001
Diseases of the sensory organs	56 (1.1)	182 (0.9)	0.17
Cardiovascular diseases	33 (0.7)	104 (0.5)	0.23
Respiratory diseases	215 (4.4)	677 (3.4)	0.002
Digestive system diseases	99 (2.0)	304 (1.5)	0.02
Skin diseases	29 (0.6)	67 (0.3)	0.01
Rheumatological diseases	51 (1.0)	126 (0.6)	0.003
Genitourinary diseases	51 (1.0)	199 (1.0)	0.87
Neonatal and congenital diseases.	158 (3.2)	397 (2.0)	<0.001
Pregnancy related diseases	29 (0.6)	155 (0.8)	0.15
Injury, poisoning and external causes of morbidity	158 (3.2)	475 (2.4)	0.002
Abnormal findings not classified otherwise	153 (3.1)	388 (2.0)	<0.001
Contacts with health services not classified above	1,049 (21.4)	4,267 (21.7)	0.58

*If not stated otherwise data is number and percentage of MD patients or individuals from the population cohort.

8,126 siblings (5,338 full siblings (65.7%)) of MD patients and 31,140 siblings (22,181 full siblings (71.2%)) of individuals from the population cohort were available for analysis. The median age of the siblings of MD patients at index date was 8.9 years (IQR 0.6–16.9) and 8.7 years (IQR 1.3–16.9) in the siblings of the individuals from the population cohort. 4,239 (52.2%) of the siblings of the MD patients were males compared to 16,079 (51.6%) of the siblings of the individuals from the population cohort.

### All-Cause Mortality

**Figure 1:**
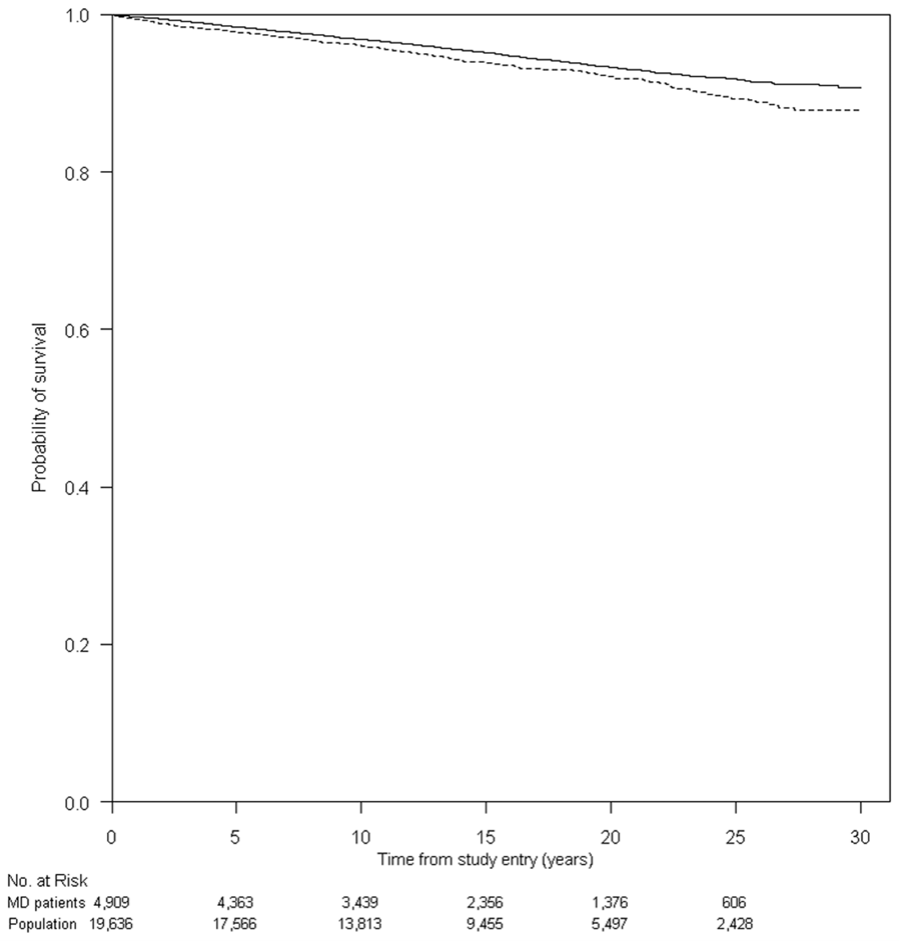
Kaplan-Meier survival curves of patients with meningococcal disease (MD) (dotted line) and individuals from the population cohort (full line).

312 (6.4%) MD patients and 985 (5.0%) individuals from the population cohort died in the observation period (Table 2). Figure 1 presents Kaplan-Meier survival curves for MD patients and corresponding individuals from the population cohort. The MRR for patients with MD was 1.27 (95% CI, 1.12–1.45), adjusted MRR 1.21 (95% CI, 1.06–1.37) (Table 2). MD patients were at increased risk of death throughout the study period and we saw no major difference in MRR between genders (Figure 1 and Table 2). The impact of MD on long-term mortality was seen in all age groups, but was only statistically significant in those diagnosed with MD after 30 years of age. In a sub-analysis in which only patients and individuals from the population cohort with no inpatient admissions in the period of two years prior to the date of MD diagnosis were included, the MRR was 1.20 (95% CI, 1.03–1.40), adjusted MRR 1.28 (95% CI, 1.10–1.49). In a sub-analysis in which patients diagnosed with nervous system or genitourinary diseases in the first year after the MD diagnosis were excluded the MRR was 1.21 (95% CI, 1.06–1.39) adjusted MRR 1.16 (95% CI, 1.01–1.34).

**Table 2:** Mortality rate ratios (MRR) for MD patients compared to individuals from the population cohort.

Characteristics	Unadjusted MRR (95% CI)	Adjusted* MRR (95% CI)	MD patients who died during the study period, N	Individuals from the population cohort who died during the study period, N
Meningococcal disease (MD), all types	1.27 (1.12–1.45)	1.21 (1.06–1.37)	312	985
Gender				
Female	1.28 (1.09–1.52)	1.22 (1.03–1.44)	180	564
Male	1.26 (1.04–1.53)	1.19 (0.98–1.45)	132	421
Age of patient at MD diagnosis				
0-<15 years	1.52 (0.97–2.36)	1.38 (0.88–2.16)	27	71
15-<30 years	1.24 (0.75–2.04)	1.29 (0.78–2.14)	20	65
30-<60 years	1.48 (1.17–1.88)	1.35 (1.06–1.73)	92	260
60 years or older	1.28 (1.08–1.52)	1.22 (1.02–1.45)	173	589
Type of MD				
1 Meningococcal meningitis	1.30 (1.13–1.52)	1.31 (1.13–1.52)	229	706
2 Acute meningococcaemia	1.19 (0.89–1.59)	1.11 (0.82–1.50)	58	195
Time period after MD diagnosis				
1-<16 years	1.29 (1.12–1.50)	1.28 (1.09–1.50)	242	752
16–30 years	1.27 (1.12–1.45)	1.20 (0.97–1.67)	70	233

*Adjusted for calendar periods and for inpatient admission prior to MD.

The MRR for siblings of MD patients compared with siblings of the individuals from the population cohort was 1.17 (95% CI, 0.92–1.48), adjusted MRR 1.15 (95% CI, 0.90–1.46). In a sub-analysis including only full siblings we found a MRR of 1.14 (95% CI, 0.83–1.55), adjusted MRR 1.11 (95% CI, 0.81–1.51). Of interest, one sibling of an individual from the population cohort died from MD compared to none of the siblings of the MD patients (data not shown). For comparison we did an analysis in which we only included MD patients and individuals from the population cohort born after January 1, 1952, and found a MRR for the MD patients of 1.51 (95% CI, 1.11–2.07), adjusted MRR 1.44 (95% CI, 1.06–1.97).

### Cause Specific Mortality

**Figure 2:**
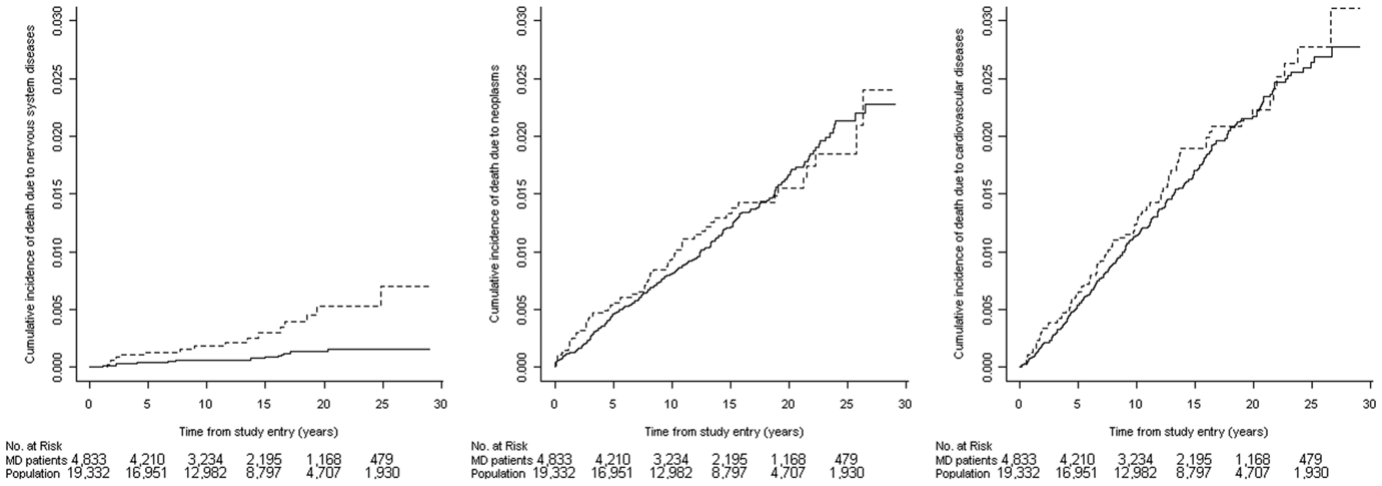
Cumulative incidences of death due to nervous system diseases, neoplasms and cardiovascular diseases for MD patients (dotted line) and individuals from the population cohort (full line). As the Danish Register of Causes of Death was only complete through 2006, patients with an index date in 2007 were not included in these analyses.

MD was associated with a statistically significant increased risk of death due to nervous system, genitourinary and digestive system diseases (Table 3 and Figure 2). An analysis of the individual causes of death due to nervous system and genitourinary diseases did not reveal any particular pattern (data not shown). The increased risk of death due to digestive system diseases was exclusively seen in patients diagnosed with MD after 30 years of age (data not shown). Further 8 of the 20 patients who died from digestive system diseases were diagnosed with alcohol abuse (Appendixes S1) at or before death compared with 9 of the 38 patients in the control group.

**Table 3:** Mortality rate ratios (MRR) for cause specific death in MD patients compared to individuals from the population cohort.

Causes of death*	Unadjusted MRR(95% CI)	Adjusted** MRR(95% CI)	Number of MD patients who died from the specific causes of death during the study period***	Number of individuals from the population cohort who died from the specific causes of death during the study period***
Infectious diseases	1.61(0.50–5.12)	1.67(0.52–5.35)	4	10
Neoplasms	1.04(0.79–1.38)	1.00(0.75–1.33)	61	235
Blood/Immune diseases	0.80(0.09–6.87)	0.53(0.06–5.07)	1	5
Endocrine diseases	1.57(0.73–3.40)	1.53(0.70–3.37)	9	23
Mental disease/drug abuse	1.13(0.54–2.37)	1.00(0.47–2.14)	9	32
Nervous system diseases	3.57(1.82–7.00)	3.15(1.59–6.23)	16	18
Cardiovascular diseases	1.09(0.85–1.38)	1.00(0.78–1.28)	84	311
Respiratory diseases	1.15(0.74–1.77)	1.09(0.70–1.70)	26	91
Digestive system diseases	2.06(1.20–3.53)	1.99(1.16–3.43)	20	39
Rheumatological diseases	2.41(0.58–10.09)	1.79(0.36–9.03)	3	5
Genitourinary diseases	5.02(1.35–18.70)	6.26(1.58–24.81)	5	4
Neonatal and congenital diseases	1.61(0.50–5.12)	1.12(0.34–3.70)	4	10
Injury and poisoning	1.32(0.86–2.03)	1.30(0.85–2.01)	28	85
Ill-defined causes	1.63(0.93–2.86)	1.65(0.94–2.90)	17	42
Unknown cause of death	0.62(0.14–2.74)	0.65(0.15–2.87)	2	13

*No causes of death due to diseases of the sensory organs, skin diseases or pregnancy related diseases were observed in the study population.

**Adjusted for calendar periods and for inpatient admission prior to MD.

***The Danish Register of Causes of Death was complete through 2006. In 2007, 23 MD patients and 62 individuals from the population cohort died and these deaths were not included in the analysis of causes of death.

MD patients were not at increased risk of death due to infections, neoplasms or cardiovascular diseases (Table 3 and Figure 2).

## Discussion

In this nationwide, population-based cohort study we found a slightly increased long-term mortality up to 30 years after patients were diagnosed with MD. The MD patients had increased risk of death due to nervous system, genitourinary and digestive system diseases of which the latter was associated with a diagnosis of alcohol abuse. We presume that the small excess mortality mainly stems from long-term sequelae and confounding.

The major strengths of the present study are its large sample size, the population-based design and the extensive and complete follow-up. The unique Danish Civil Registration System enabled us to identify a large control cohort of individuals well matched in terms of gender, age, and country of birth. Through the Danish national registers we had access to complete data on date of death, comorbidity, and causes of death and importantly, this data was obtained from the same data sources for both cohorts. The registers further allowed us to track all siblings of the study subjects.

Considering the limitations of the study we relied on register-based discharge diagnoses. Thereby the study population comprised MD patients irrespective of whether the diagnosis was confirmed by microbiological tests or exclusively based on clinical criteria. Although discharge diagnoses in general may not be entirely accurate, the registration of MD in the Danish National Hospital Register has been shown to be substantially valid [8], [15], [16]. Pre-hospital antibiotic treatment is mainly administered to the most severe MD cases [17], and may lead to decreased sensitivity of the microbiological tests performed after hospitalization. Thus, inclusion of only bacteriologically verified cases would most likely have biased the estimates of long-term mortality by exclusion of the most severe MD cases. Also, from the patient's point of view, the main interest is the long-term prognosis according to the discharge diagnosis. We therefore believe that our study, not only adds to the understanding of the medical aspects of MD, but also is of considerable relevance to the patients, who are diagnosed with MD and have a natural interest in knowing their long-term prognosis.

Due to the nature of our study with an inclusion period of 30 years and inclusion of MD patients diagnosed at all hospitals in Denmark, we did not have access to clinical and paraclinical data obtained during the hospitalizations. Our data thereby does not allow identification of clinical predictors of long-term mortality or information on neurologic sequelae in the MD patients. Also, we were not able to control for confounding from smoking including passive smoking, alcohol consumption, educational level, socioeconomic status or crowding.

Cases of MD are often categorized into septicaemia (approximately 30% in outbreaks), meningitis (10%) and mixed disease (60%) [18]. The Danish National Hospital Register allows one primary diagnosis and patients diagnosed with mixed MD are primarily registered as meningococcal meningitis. In accordance with this practice, we observed a somewhat higher proportion of patients registered primarily with meningitis (67%).

We observed a slightly increased long-term mortality in the MD patients in all age groups. To our knowledge only one previous study has examined the long-term mortality in MD patients [4]. The authors found an increased risk of death during the first four years following diagnosis of bacterial meningitis, whereas the risk of death declined to that of the background population from the fifth year of discharge. The study, however, only included 356 MD patients, had substantially shorter follow-up and pre-existing comorbidity was not accounted for.

We find it unlikely that comorbidity diagnosed prior to MD explains our findings, as the analysis, which only included patients not hospitalized prior to their MD diagnosis demonstrated essentially the same increased mortality as found for the complete MD study population. We were however only able to adjust for comorbidity in case it led to hospitalization and these analyses are probably hampered by unmeasured and residual confounding.

We observed an increased risk of death due to nervous system diseases, which is probably a consequence of the neurologic sequelae known to occur after MD [7], though the pre-existing higher admission rate for nervous system diseases before the diagnosis of MD may also have lead to increased risk of neurologic death. Nervous system diseases are known to lead to urological complications and we presume, that the increased risk of death due to genitourinary diseases could be related to this phenomenon, or to persistent kidney damage after MD sepsis-associated acute renal failure [19]. Exclusion of the patients diagnosed with nervous system or genitourinary diseases in the first year after the MD diagnosis reduced the estimated MRR, which further indicates that the increased mortality in the MD patients partly stems from sequelae in these organ systems. Several of the patients, who died from digestive system diseases, were diagnosed with alcohol abuse. Whether this was due to an increased risk of MD in alcoholics or an increased risk of alcohol abuse after MD could not be established in the actual study.

Some inherited immune deficiencies predispose to MD, but the prevalence of these conditions among patients with MD is low [1], [20]. Likewise we did not observe any increased risk of death due to infections in the MD population. However, not all infections cause death and the fact that the MD patients had more inpatient admission for infectious diseases in the two years prior to the diagnosis of MD than the population cohort keeps the possibility of a specific immune defect open.

A point of interest revealed in our study was a trend towards a slightly increased long-term mortality in siblings of MD patients, although these associations were not statistically significant. Factors other than the pathogenicity of the MD infection per se likely contribute to the increased long-term mortality in MD patients. In the present study we could not determine whether these factors are of a genetic or environmental nature.

We conclude that patients diagnosed with MD have an increased long-term mortality, but the excess mortality is small. The MD population has no increased risk of death due to infections, neoplasms or cardiovascular diseases, but suffers from increased mortality mainly due to nervous system diseases. With the actual findings, patients treated for meningococcal disease can be reassured that their long-term mortality differs only slightly from that of the general population.

## Supporting Information

Appendixes S1 (0.02 MB DOC)
