# Supplementary material for: Long-Term Mortality in Patients Diagnosed with Meningococcal Disease: A Danish Nationwide Cohort Study
Source: PLoS One. 2010 Mar 12;5(3):e9662. doi: 10.1371/journal.pone.0009662 (PMC2837384; doi:10.1371/journal.pone.0009662)
Supplement: Appendixes S1 — (0.02 MB DOC) [file pone.0009662.s001.doc]

**APPENDIX 1**

Infections of the central nervous system were defined by the following ICD-8 codes for the years 1977-1993. For bacterial meningits the codes were 320.00-320.99; Listerial meningiits 027.01; Meningococcal infection 036.00-036.99; Aseptic meningitis due to enterovirus 045.00-045.99; Varicella meningitis/encephalitis 052.01; Zoster meningitis/encephalitis 053.02 and Herpesviral meningitis/encephalitis 054.03.

Infections of the central nervous system were defined by the following ICD-10 codes for the years 1994-2007. For inflammatory diseases of the central nervous system the codes were G00.0-G01.9; Listerial meningitis and meningoencephalitis A32.1; Meningococcal infection A39.0-A39.9; Unspecified viral encephalitis A86; Enteroviral meningitis A87.0; Adenoviral meningitis A87.1; Lymphocytic choriomeningitis A87.2; Other viral meningitis A87.8; Viral meningitis, unspecified A87.9; Herpesviral meningitis B00.3; Herpesviral encephalitis B00.4; Varicella meningitis B01.0; Varicella encephalitis B01.1; Zoster encephalitis B02.0; Zoster meningitis B02.1; Zoster with other nervous system involvement B02.2.

**APPENDIX** **2**

The eighteen categories of primary causes of death were specified by ICD-8 codes for the years 1977-1993 and ICD-10 codes from 1994 to 2006. For infectious diseases, the codes were 000-134.99/A00-B99; neoplasms 140-239.99/C-D48; blood/immune diseases 280-289.99/D50-D89; endocrine diseases 240-279.09/E00-E90; mental diseases/drug abuse 290-315/F00-F99; nervous system diseases 320-358.09/G00-G99; diseases of the sensory organs 360-389.99/H00-H59; cardiovascular diseases 390-458.99/I00-I99; respiratory diseases 460-519.99/J00-J99; digestive system diseases 520-577.99/K00-K93; skin diseases 680-709.99/L00-L99; rheumatological diseases 710-738.09/M00-M99; genitourinary diseases 580-629.99/N00-N99; neonatal/congenital disorders 740-779.99/P00-Q99; pregnancy related diseases 630-678.09/O00-O99; injury/poisoning 800-999/S00-T98,V,W,X,Y; ill-defined causes 780-796.99/R00-R99 and no cause of death reported.

**APPENDIX** **3**

The eighteen categories of primary discharge diagnoses were specified by ICD-8 codes for the years 1977-1993 and ICD-10 codes from 1994 to 2006. For infectious diseases, the codes were 000-134.99/A00-B99; neoplasms 140-239.99/C-D48; blood/immune diseases 280-289.99/D50-D89; endocrine diseases 240-279.09/E00-E90; mental diseases/drug abuse 290-315/F00-F99; nervous system diseases 320-358.09/G00-G99; diseases of the sensory organs 360-389.99/H00-H59; cardiovascular diseases 390-458.99/I00-I99; respiratory diseases 460-519.99/J00-J99; digestive system diseases 520-577.99/K00-K93; skin diseases 680-709.99/L00-L99; rheumatological diseases 710-738.09/M00-M99; genitourinary diseases 580-629.99/N00-N99; neonatal/congenital disorders 740-779.99/P00-Q99; pregnancy related diseases 630-678.09/O00-O99; injury/poisoning 800-999/S00-T98,V,W,X,Y; abnormal findings not classified otherwise 780-796.99/R00-R99 and contacts with health services not classified above Y00-Y95.

**APPENDIX** **4**

Alcohol abuse was defined by the following ICD codes: ICD-8: 291.00-291.99, 571.09, 571.10, 303.00-303.89, 303.91-303.99 and ICD-10: K70.0-70.9, F10.2-10.9, G31.2.
